# Advice for authors from the editors of *Perspectives on Medical Education*: Getting your research published

**DOI:** 10.1007/s40037-018-0483-0

**Published:** 2018-11-28

**Authors:** Lara Varpio, Erik Driessen, Lauren Maggio, Lorelei Lingard, Kalman Winston, Kulamakan Kulasegaram, Alisa Nagler, Jennifer Cleland, Johanna Schönrock-Adema, Elise Paradis, Anne Mette Mørcke, Wendy Hu, Margaret Hay, Martin G. Tolsgaard

**Affiliations:** 1 Uniformed Services University of the Health Sciences, Bethesda, MD USA; 2 Editors Perspectives on Medical Eduction, https://www.springer.com/education+%26+language/journal/40037?detailsPage=editorialBoard

Doing good health professions education (HPE) research is hard. Getting that research published is, arguably, harder still. The number of high quality manuscripts submitted to HPE’s academic journals far exceeds the number of pages available. It is not surprising, then, that journals such as *Perspectives on Medical Education (PME), Academic Medicine, Medical Teacher, *and *Medical Education* reject over 70% of incoming manuscripts. To compete for inclusion in an academic journal, researchers need to be both accomplished scientists *and* rhetorically skilled authors.

Fortunately, there are many publications that offer sound advice to authors, explaining why papers get rejected, [1–4] and suggesting tips on how to craft powerful manuscripts [5–7]. While this sage counsel can help you write more persuasive manuscripts, there is another community that should advise HPE scholars: journal editors. Many authors are naïve to the editorial considerations that factor into decisions to accept or reject manuscripts. Without such insights, you risk remaining unaware of important considerations influencing the outcome of your journal submissions.

As researchers, authors, and editorial board members for *PME, *we have assembled key insights gained from our experiences. While not the definitive list of tips that every journal editor adheres to, our advice reflects the personal experiences, values, and interdisciplinary traditions of our editorial team. In this editorial, we offer our insights in hopes of improving authors’ chances of having their manuscripts accepted for publication in HPE journals.

## Keep context in mind

Authors often neglect to communicate the implications of their research for readers outside of their own institution or research team. While the functioning of a new drug could differ relatively little (or not at all) in different populations, the same is not the case with HPE research. This has at least two implications for editors evaluating research articles.

First, if anyone outside of your institution or research team is to benefit from study results, you need to position the study within a broader context and/or a theoretical framework. The importance of theory and transferability to high-quality research has been emphasized repeatedly in our research community [1–3, 8] but its absence remains one of the main reasons for rejection.

Second, a *good* HPE research study is not simply one that displays strong methodological rigor. Instead, a *good* HPE research study also provides sufficient detail about what was done, where, why, and to whom so that the reader can understand the relevance of the findings to their context. Just like theory, the context and conditions of the study may be used as a lens through which the study results are understood and interpreted [2].

## Join a scholarly conversation

Editors not only judge individual manuscripts, they curate scholarly conversations. This plays a role in determining which of the many *good* submissions go out for review, and which are desk-rejected because they do not fit with the scholarly conversations the journal is curating. Knowing this, you should clearly signal how your work is relevant to the scholarly conversations taking place in your targeted journal, and more generally within the field around that specific topic [9].

The scope of a paper’s contribution to that conversation is also important. Papers that focus on a narrow niche may be central to the journal’s mandate or, by contrast, may attract only a small subset of readers. If the paper is focused on a narrow niche, or a very specific local context, you should be clear how it may benefit a more general readership, or consider whether the paper may be better placed in more specialist or local publications. Conversely, a paper with broad appeal must still fit the journal’s mandate, but might attract new readers, especially if it brings into the conversation perspectives from diverse audiences and contexts that may be less frequently included. Indeed, because conversations with a variety of contributing voices produce richer, more robust insights, editors may consider whether a paper presents an opportunity to engage under-represented groups (e. g., students, or scholars from the developing world) in scholarly conversations.

## Connect with the right audience

Health professions educators come together into a broad, inclusive community of researchers and practitioners who engage with their communities in different ways. Editors expect you to determine the message that will connect with your chosen audience, and the most effective way to engage that audience. So, before submitting, you should think laterally—which audience do I want to address? For whom will my message make a difference, by adding something new, changing practice, or influencing the direction of advances?

While all academic journals disseminating HPE scholarship publish research papers, there are a myriad of other manuscript types within each journal. Each type appeals to different audiences within our community. For instance, if you are looking to connect with educators who develop and implement educational innovations, consider *PME’s* Show & Tell. If you want to present a new perspective on an educational topic or a new idea to the HPE community, consider *PME*’s Eye Openers. If you want to share lessons learned from research and/or innovation experiences that didn’t go as planned, consider *PME*’s Failures/Surprises. Depending on the story you wish to tell, be sure to submit the paper for consideration in the right manuscript category so that you reach your target audience. When in doubt, many editors welcome pre-submission queries from authors to advise on the most appropriate article type and whether their journal could be a good fit for the paper in question.

## Consider the manuscript’s news value

The excess of submitted papers requires editors’ decisions to factor in considerations other than mere study quality. One of the factors editors must consider is the manuscript’s *news value*: Does the paper help to move the thinking in our field forward in an important way? Unfortunately, there are no clear-cut criteria for determining a paper’s news value, but there are some common questions that regularly inform this consideration: Is the topic new and relevant for the field? Does the manuscript bring an innovative and/or important perspective to an ongoing discussion? Are the study methods novel and relevant to other researchers? Is the paper giving voice to an underrepresented group or perspective? Is the topic timely (i. e., a *hot *topic)? Is there something in the manuscript that elicits a powerful reaction? You can gauge the news value of your paper by thinking about the audience: Ask yourself, is the message of the paper important enough to get the attention of your busiest colleagues?

It can, however, be challenging to read your paper through the news value lens. Often there were very legitimate reasons to do a study that have no direct relation with the potential news value of the resulting paper. Therefore, it makes sense to ask yourself from the start of the research project the news value questions: Who will be my audience? Why will they read my paper? Is the potential readership large enough to warrant publication in the target journal? Will readers cite the manuscript in their own work? Will they recommend it to others? Will it stimulate a new line of research? Will it affect policy and practices?

## Address the perspectives of all the manuscript’s reviewers

If an editor decides that your manuscript might be worthy of inclusion in the journal, she sends it out for review. The editor must then determine who should be invited to be a reviewer. There are several questions that must be answered by the reviewers, including: Is the manuscript methodologically rigorous? Is it contributing to current aspects of conversations on the topic? Is it relevant to potential readers outside of the author’s local context? To do this, editors will often try to secure three different types of reviewers: a methods expert (e. g., a researcher skilled in the methodologies and methods used in the study), a topic expert (e. g., a researcher who is actively publishing on the topic being addressed in the manuscript), and a potential user (e. g., a person who could implement the findings in their local context).

Given these different reviewer perspectives, you are well advised to ensure your manuscript addresses all three areas of focus. Some simple questions can help guide you in this work:

1. Methods: Have you demonstrated an in-depth understanding of the methodology and methods being used in the manuscript? How did you establish rigor? Have you justified your methodological and methods choices and utilized them appropriately?
2. Topic: Who is currently contributing to the conversation on the topic being addressed in your manuscript? How are you adding to this conversation? Are your citations sufficiently current?
3. Other users: Could a researcher or practitioner from a different institution use your findings? What theory have you used to support the transferability or generalizability of your conclusions? Have you established why your findings are relevant to others?

## Don’t let an editor be the first person to review the manuscript

While finding methods-, topic-, and user-focused reviewers is the ideal, it is an ideal that is hard to achieve. One of the biggest challenges facing editors is finding expert reviewers willing and able to volunteer a high quality review for a manuscript. While there is a large pool of authors vying to be included in the pages of a journal, there are considerably fewer reviewers volunteering to offer peer reviews. Given that, if editors spot problems in the manuscript—e. g., weaknesses in the methods, or faulty logic underpinning the paper’s argument—they may opt simply to reject the manuscript as opposed to sending it out for peer review. If your paper will not stand up to scrutiny, it is best for the editors to conserve the limited resource of peer-review time for those manuscripts that are ready for sharp-eyed peers.

A journal editor should never be the first person to review and critique your manuscript. Instead, consider developing a community of peer reviewers for manuscripts, perhaps even engaging in-group peer review yourself. Group peer review allows for the leveraging of individual group members’ skills and experiences, and it fosters faculty development as members learn from one another. Scholars who have piloted group peer review have praised the high-quality feedback that is developed by the group [10, 11]. Critique from colleagues prior to submission can greatly improve the quality of your manuscript, increasing the likelihood that editors will not identify problems that lead to an editorial desk rejection.

## Be vigilant about questionable research practices

The prevalence of, and thus concerns about, questionable research and publishing practices is on the rise in HPE [12]. Editors are becoming well-versed in identifying salami slicing, honorary authorship, failure to comply with ethical review board guidelines, and a host of other unethical practices. While most researchers can recognize and avoid flagrant ethical violations (e. g., plagiarism, data fabrication), it can be difficult in some cases to know if one is operating in an ethical grey zone.

For example, salami slicing is the practice of breaking apart a single research study into smaller pieces to increase the number of publications. At first glance, it seems obvious that salami slicing is a practice to avoid. However, re-analyzing datasets can be relevant when engaging in research programs where individual datasets can be analyzed multiple times, through a variety of theoretical lenses, and/or in combination with other datasets. Thus, editors are required to both keep a keen eye out for the unethical practice of salami slicing, but also being aware that analyzing the same data set multiple times is sometimes required in programs of research. Authors can help editors make these judgements. Before writing another paper using the same dataset, ask yourself: Will this contribution be significantly different from others made with this data? Will readers of the multiple manuscripts in the program of research clearly see how this work is novel [13]?

Transparency is key when it comes to questionable research practices. Make the reviewers’ and editors’ jobs easier by addressing research practices upfront in your cover letter and again in the introduction or methods section of the manuscript. In the case of salami slicing, you can explain how the manuscript reports on novel work, how it relates to a different and important research question, how it is related to existing work and is not overlapping or duplicating that work. Cite the previous publications in the program of work so that the editors can confirm that salami slicing was avoided. Candour and clarity on these issues helps editors immensely.

## Use the cover letter

We know that by the time you have finished writing your paper, formatted the references, and tamed the manuscript’s word count down to size, you are ready to move on to other projects. However, before submitting, you still need to draft a cover letter. It might be tempting to skip it or to cut and paste chunks of the abstract into letter format, but don’t! For editors and reviewers, the cover letter is the first interaction with your submission. Use this opportunity to make a good first impression by being professional (e. g., use proper business correspondence format, correctly address the editor), be concise (keep it to a page or less), ensure grammatical correctness (enlist a trusted colleague as a proof-reader), and adhere to your target journal’s cover letter requirements (check the author instructions).

Leverage the cover letter as your first chance to market your manuscript to the editors and convince them why they need to publish your article in their journal. Be specific; describe how your manuscript aligns with the journal’s scope, would be of interest to its readers, and how it expands on current conversations in the field.

## Read a lot, and broadly

An editor’s chief responsibility is to come to a well-reasoned, clearly thought-out judgment about the quality and importance of the manuscript they are adjudicating. However, not every manuscript assigned to an editor sits squarely within her area of expertise. Editors often do additional reading to understand all aspects of the submitted work. For example, if she is unfamiliar with your research methodology, she will find an expert reviewer with that expertise. Moreover, she will likely do some background reading herself about the methodology to find out about it, rather than depending wholly on a review. The editor is also likely to look in the journal itself to see if any papers have been previously published using the same methodology because this can be a useful yardstick of what has been evaluated and included in the journal previously. In sum, editors spend a lot of time reading the literature in order to be able to responsibly appraise your manuscript.

Likewise, it is to your advantage to be well-read. Since editors engage in considerable periphery reading to better understand and situate manuscripts in the relevant literatures, you too should ensure that you are engaging in such readings, and citing them. One of the best tips for success--for editors and authors alike--is to read copiously and widely.

## Recognize the thoughtful judgment that went into the editorial decision

Reviewers of a manuscript often disagree: one will love the manuscript, one will find many serious flaws, and one will be indifferent. This is the reality that editors must negotiate for most manuscript decisions. As a result, most editorial decisions are based on reviewer feedback in combination with the editor’s own appraisal of the manuscript. The editor must make decisions about how much weight she attaches to any reviewer’s remarks and then combine the opinions of all parties to (1) make a well-considered decision about acceptance or rejection of your paper; and (2) highlight which revisions are essential to improve the manuscript (either for resubmission to this journal or to submit to another journal). This process of coming to a decision and writing a decision letter requires professional judgment.

When you receive editorial decisions, it is easy to focus on the positive reviews and feel that they outweigh any negative comments. It can be frustrating if the editor has rejected your work despite many supportive observations from reviewers. But editorial decisions are not taken lightly. When you receive negative feedback about your manuscript, let the news sit with you for a few days before making revisions or submitting the work to a new journal. Ask others who are not involved with the paper to interpret the instructions from the editor and reviewers. Take into consideration that in some cultures (e. g., Canada, UK), reviewers tend to wrap their critical comments with supportive and polite remarks. A reviewer’s remarks can have different connotations, depending on the reviewer’s culture.

When an editor asks for revisions, consider that request carefully and do the work the editor asks of you. A ‘major revision’ should be taken seriously and requires you to engage in and demonstrate major work on your paper.

## Be a peer reviewer

Volunteering to be a peer reviewer is an excellent way to become a better author. Editors endeavour to provide constructive feedback in their editorial decision letters, even if the decision is a rejection. In this way, the editor shows authors that she has paid attention to their work and engaged with it thoughtfully. Editors generally use their editorial roles to serve the community, sharing expertise and integrating the feedback provided by reviewers to guide authors with directed critique. This guidance can help you to improve your ongoing research and writing.

Most academic journals now send reviewers the decision letter given to the authors because this information is *highly valuable *to the reviewer. As a reviewer you learn which of your critiques resonated with the editor, which concerns you missed in your reading, and the reasoning that guided the editor’s final decision. These glimpses into the ways in which editors make *accept* versus *reject* decisions offer you insights into what makes for a successful submission.

## Cure zombie papers with tough love

Unfortunately, editors have to hand out rejections regularly. Rejections can demotivate you and the editor alike—it is unpleasant for everyone involved. Moreover, a rejection does not necessarily mean that your paper is of poor quality: editors reject good manuscripts for a variety of reasons.

Consequently, many of us have zombie papers: manuscripts that contain a good idea or an interesting finding for which you just can’t seem to find a publisher, so it lurches along on your publication ‘to do’ list, never dying and never really coming to life. These manuscripts should not be abandoned, unless hindsight tells you the study was just not designed properly. Often, the feedback from reviewers and editors can help you to describe the good idea more persuasively, or to frame the interesting data more appropriately. Zombie papers can only find publication homes if the authors are willing to treat their manuscripts as objects to be revised and refined over and over again. Too often, authors treat their papers as precious things that should not be sullied by the changes suggested by others. Although you should be passionate about your research and innovations, you are well-advised to be a little hard-hearted about your writing. A little tough love usually improves a manuscript.

## Conclusion

The decision to accept or reject a manuscript is shaped by a multitude of factors. The tips we provide are subjective and by no means universally applicable since they reflect the opinions, values, perspectives, and research traditions of our team of editors. That said, we suggest that a key lesson for authors to glean from our advice is that methodologically rigorous research is not in-and-of itself sufficient reason for a manuscript to be accepted for publication in academic journals. A good HPE study is not merely about having a great research idea; instead, it is about telling a story that adds something to our general knowledge body. The tips above highlight that considerable effort is needed when researching the relevance of your research project and when placing it within the current body of literature to further the field of HPE. We hope that the advice we offer will help you to successfully publish your research.

## Disclaimer

The views expressed herein are those of the authors and not necessarily those of the Department of Defense or other federal agencies.
